# A Novel Convolutional Neural Network for Explainable Diabetic Retinopathy Detection and Grade Identification

**DOI:** 10.3390/s26082510

**Published:** 2026-04-18

**Authors:** Simona Correra, Valeria Sorgente, Mario Cesarelli, Fabio Martinelli, Antonella Santone, Francesco Mercaldo

**Affiliations:** 1 Department of Medicine and Health Sciences “Vincenzo Tiberio”, University of Molise, 86100 Campobasso, Italy; antonella.santone@unimol.it; 2 Department of Engineering, University of Sannio, 82100 Benevento, Italy; mcesarelli@unisannio.it; 3 Institute for High Performance Computing and Networking, National Research Council of Italy (CNR), 87036 Rende, Italy; fabio.martinelli@icar.cnr.it

**Keywords:** artificial intelligence, deep learning, convolutional neural network, classification, diabetic retinopathy

## Abstract

Diabetic retinopathy represents one of the leading causes of blindness worldwide, making early diagnosis essential for effective clinical intervention. We propose an explainable method aimed at automatically identifying the severity levels of diabetic retinopathy in retinal images using deep learning. The proposed method considers several convolutional neural network architectures, i.e., VGG16, StandardCNN, ResNet, CustomCNN, EfficientNet, MobileNet, and a novel architecture, i.e., FGNet, specifically designed and developed by the authors for diabetic retinopathy detection. The proposed network achieves an accuracy of 0.75 when trained for 10 epochs and 0.71 for 20 epochs. Explainability behind model prediction is further supported through Gradient-weighted Class Activation Mapping, providing visual insight into the learned decision-making process and potentially supporting early clinical assessment.

## 1. Introduction

The global rise of diabetes has brought increasing attention to one of its most debilitating complications: Diabetic Retinopathy (DR). As a matter of fact, diabetes prevalence continues to climb due to sedentary lifestyles, dietary shifts, and an aging population; the number of patients affected by DR is expected to reach nearly 161 million by 2045 [1]. Currently, between 30% and 40% of diabetic individuals develop retinal damage, making DR a major public health concern and a leading cause of vision impairment worldwide.

DR emerges from chronic hyperglycemia, which progressively compromises the integrity of the retinal microvasculature. Early manifestations include microaneurysms, hemorrhages, venous abnormalities, lipid deposits, and focal areas of nonperfusion. If these pathological changes remain untreated, they may evolve into Diabetic Macular Edema (DME) or into advanced stages of DR, often resulting in irreversible vision loss [2].

Clinically, this disease is categorized into non-proliferative (NPDR) and proliferative (PDR) forms [3]. NPDR represents the initial phase, typically characterized by limited microvascular damage and preserved visual acuity. As ischemic stress increases, the retina releases vascular endothelial growth factor (VEGF), which promotes the formation of abnormal vessels. This process marks the transition to PDR, a severe condition marked by extensive neovascularization, vascular leakage, and ischemia. These alterations can lead to tractional retinal detachment, vitreous hemorrhage, and profound visual impairment.

Management strategies reflect this progression. While metabolic control of glucose, lipids, and blood pressure can slow NPDR, advanced disease stages often require more invasive treatments. Laser photocoagulation, intravitreal corticosteroids, and anti-VEGF agents are commonly used interventions, although their repeated administration may cause side effects such as inflammation, changes in intraocular pressure, and retinal scarring [3]. To support clinical decision-making, standardized systems such as the ETDRS classification and the ICDR Severity Scale provide structured criteria for assessing disease severity [2]. The effectiveness of automated diagnostic systems is strongly influenced by the imaging devices used for retinal data acquisition [4]. In particular, digital fundus cameras, which are widely adopted in large-scale screening programs, provide two-dimensional representations of the retina that enable the identification of lesions such as microaneurysms and hemorrhages. However, variations in image quality, illumination, resolution, and acquisition protocols may significantly affect the reliability and generalization capability of machine learning models [5,6]. Advanced imaging modalities such as Optical Coherence Tomography (OCT) have been introduced, providing high-resolution cross-sectional visualization of retinal layers and enabling the detection of subtle structural alterations that are not visible in standard fundus images [7]. Despite these advantages, most large-scale datasets used for deep learning training are still based on fundus imaging, thus highlighting the need for robust models capable of handling heterogeneous data acquired from different sensors and clinical settings [4]. Convolutional Neural Networks (CNNs) and, more generally, machine learning approaches demonstrate interesting accuracy in identifying lesions and grading DR directly from retinal fundus images [8,9,10]. Beyond imaging, predictive solutions integrating continuous glucose monitoring data have been explored using techniques such as Linear Discriminant Analysis, Support Vector Machines, and Random Forests [11].

The main limitation that limits the adoption of these approaches in daily clinical practice is the total lack of explainability, meaning the clinician is unable to understand the results generated by a model, an aspect that severely limits the confidence of the clinician in using these methods to support diagnosis.

Starting from the considerations, in this paper, we propose an explainable approach for diabetic retinopathy classification. In a nutshell, the proposed method considers a set of CNNs (one of these, i.e., FGNet developed by the authors specifically for DR detection task) aimed to identify DR with the related grade, while heatmap-based explanations are provided to highlight the retinal regions and pathological patterns that are symptomatic of the DR disease from the model point of view. In addition to the FGNet, i.e., the network designed and developed by the authors, we also consider other CNNs, i.e., VGG16, StandardCNN, ResNet, CustomCNN, EfficientNet, MobileNet, and DenseNet.

The remaining of the paper is organized as follows: Section 2 reviews related work on DR detection, Section 3 details the method, and Section 4 presents the experimental setup and results. Finally, Section 5 summarizes the findings and outlines directions for future research plans.

## 2. Related Work

Machine learning and, more specifically, deep learning have emerged as powerful methods in numerous areas of bioengineering and medicine, providing substantial contributions to the early diagnosis of complex diseases such as cancer and other pathological conditions.

As a matter of fact, one of the most impactful applications lies in medical image analysis, such as the automated interpretation of retinal images for the diagnosis of ophthalmic diseases such as DR. In fact, if not detected early, DR can lead to irreversible vision loss or blindness.

In this direction, numerous studies have investigated machine learning and deep learning approaches for the automated detection of DR from retinal fundus images. A comparative summary of these approaches is provided in Table 1 and presented below.

For instance, a recent review by Zhu et al. [12] analyzed 94 studies on deep learning–based DR classification. The authors highlight transfer learning as a common strategy to address limited data availability and identify CNN architectures such as VGG and ResNet as the most widely used models. In particular, five-class DR classification experiments report accuracies of 0.87 using VGG16 and up to 0.96 with DenseNet121. Despite the high performance achieved by deep learning models, the study emphasizes the need for improved interpretability and clinical reliability.

Another study by Abushawish et al. [13] reviews the evolution from traditional machine learning approaches to advanced CNN-based architectures, analyzing 26 pre-trained models from different architectures, including DenseNet, ResNet, VGG, and EfficientNet. The authors also investigate hybrid models that combine multiple CNNs and apply Gaussian filtering as a preprocessing step. Experimental results show that single models such as ResNet50 achieve accuracies of approximately 0.80, while hybrid architectures, including DenseNet121–ResNet50 combinations, reach accuracies exceeding 0.98. Model interpretability is further addressed through Grad-CAM visualizations to support clinical decision-making.

Gulshan et al. [14] developed and validated a CNN for the automatic detection of DR from fundus photographs. The proposed approach employed a deep learning architecture trained end-to-end on a large dataset of labeled retinal images, leveraging transfer learning and extensive data augmentation strategies. The model was evaluated on two independent test datasets, namely EyePACS-1 and MESSIDOR-2, to assess its generalization ability. Experimental results demonstrated exceptional performance, achieving an AUC of 0.991 on EyePACS-1 and 0.990 on MESSIDOR-2 for DR detection. These results highlighted the potential of deep CNN-based systems to achieve performance levels comparable to those of experienced ophthalmologists.

In another study [10], the authors present a CNN-based strategy for DR analysis from ocular angiography, which integrates disease detection and severity classification. The proposed models obtain accuracies of 0.98 and 0.91, respectively. Visual explanation is provided through class activation mapping, enabling localization of pathological regions.

Authors in [15] investigates machine learning approaches for DR classification by considering handcrafted features. A method leveraging radiomic features extracted from retinal fundus images and different classifiers was proposed, achieving an accuracy of 0.846 on a public dataset, along with competitive precision and recall values; however, the approach does not incorporate explicit explainability mechanisms to support the interpretation of the model decisions.

Mohanty et al. [16] introduce a hybrid framework that combines VGG16 for feature extraction with an XGBoost classifier, as well as an end-to-end DenseNet121 model. Experimental results show that the hybrid VGG16–XGBoost approach achieves an accuracy of 0.80, while the DenseNet121 model attains a significantly higher accuracy of 0.97, outperforming other existing methods evaluated on the same dataset.

In addition to algorithmic advances, the type and quality of the imaging sensors used for data acquisition play a critical role in model performance. Variations in fundus camera resolution, field-of-view, illumination, and acquisition protocol across different clinics can introduce heterogeneity in public datasets, affecting both training and generalization of deep learning models [4,17]. Moreover, advanced modalities such as OCT and multimodal imaging provide richer structural information of the retina, allowing the detection of subtle features not visible in standard fundus photography [18]. Despite these advantages, most large-scale DR datasets remain based on fundus images, highlighting the importance of developing robust models capable of handling heterogeneous data from different sensors and settings.

Overall, the existing literature demonstrates that deep learning–based approaches are highly effective for the automated detection and classification of DR from retinal images. However, despite the promising performance reported across different studies, several challenges remain open, including reliable multi-stage classification, robustness across heterogeneous datasets, and the development of diagnostic systems that can effectively support early disease detection in clinical practice. In this context, the proposed method proposes and investigates the use of CNN architectures for classifying retinal images across different stages of DR, with the aim of developing a computer-aided diagnostic methodology that supports physicians in the early diagnosis of the disease.

## 3. The Method

**Figure 1:**
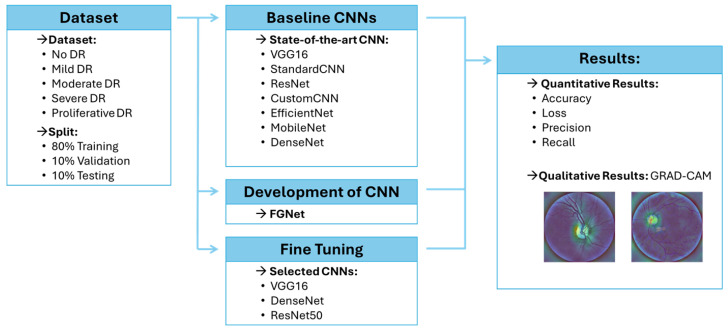
The workflow of the proposed method, highlighting the main phases of data processing and model evaluation.

In this section, we present the method we designed and developed for explainable DR detection. The workflow of the proposed method is shown in Figure 1: it consists of three main phases.

The first step of the workflow consisted of training several CNNs after selecting the target dataset. This phase is intended to assess the classification performance of each model and to generate Grad-CAM visualizations in order to support the explainability of the obtained predictions.

In the second phase, a novel network architecture, i.e., *FGNet,* is designed and developed. The FGNet model is subsequently trained and evaluated on the same dataset, and the corresponding Grad-CAM heatmaps were produced to analyze the obtained performance.

Finally, in the third step, only the networks achieving the best results in terms of classification accuracy and Grad-CAM quality are subjected to a fine-tuning procedure.

### 3.1. The CNN Models

In the first stage of the proposed workflow, several state-of-the-art convolutional neural network architectures were selected to enable a comparative evaluation of their performance, as described below. The following CNNs are considered:

- VGG16, a deep CNN characterized by an architecture composed of multiple stacked convolutional layers followed by pooling and fully connected layers, and widely adopted as a reference model in image classification tasks;
- StandardCNN: An architecture composed of a sequential arrangement of convolutional layers, pooling operations, and fully connected layers, and this network was implemented by the authors of the referenced paper [19].
- ResNet: A CNN architecture that introduces residual connections to facilitate the training of deep models and to achieve improved performance as depth increases.
- CustomCNN: Another custom-designed CNN developed by the authors, as for StandardCNN, for comparative purposes.
- EfficientNet: A CNN architecture based on a compound scaling strategy that simultaneously scales network depth, width, and input resolution, achieving high accuracy with a reduced number of parameters and lower computational cost.
- MobileNet: A lightweight CNN specifically designed for mobile and embedded devices. The architecture employs depthwise separable convolutions to significantly reduce computational complexity and model size while preserving competitive performance.
- DenseNet: A densely connected CNN in which each layer receives as input the feature maps from all preceding layers. This connectivity pattern promotes feature reuse, improves information flow, and alleviates the vanishing gradient problem.

For each architecture, several training configurations were explored by varying the learning rate and batch size, while performance was quantitatively analyzed using loss, accuracy, precision, and recall metrics.

The developed network, i.e., FGNet, is composed of two main components: a convolutional block and a classification block. The convolutional block included three convolutional layers (Conv2D) with 32, 64, and 128 filters of size 3 × 3, each followed by a ReLU activation function.MaxPooling2D layers were interleaved between the convolutional layers to progressively reduce the spatial resolution of the feature maps while preserving the most relevant information. The classification block consisted of a Flatten layer followed by three fully connected (Dense) layers with 256 and 128 neurons. The first two Dense layers employed ReLU activation functions, while the final Dense layer used a softmax activation function to produce class probability scores. Three Dropout layers were interposed between the Dense layers, each randomly deactivating 50% of the neurons during training in order to reduce overfitting and enhance the generalization capability of the model.

FGNet was designed as a lightweight CNN aimed at achieving effective classification performance while maintaining a reduced computational footprint. The network structure is defined to limit architectural complexity, allowing efficient feature extraction and making the model suitable for real-world, practical, and scalable deployment.

The results obtained will provide an overview of model performance across architectures and training strategies, highlighting the comparative strengths of each approach.

### 3.2. Performance Metrics

Model performance in DR detection is evaluated using quantitative metrics, presented below, commonly adopted in multiclass classification tasks. Validation loss, which measures the discrepancy between predicted and ground-truth labels, and Accuracy were considered the primary performance indicators.

In particular, Accuracy is the measure of correctly classified images relative to the total number of samples in the dataset. It is defined as:

(1) $$Accuracy=\frac{TP+TN}{TP+TN+FP+FN}$$

with the following notation, computed for each class using a one-vs-rest (OvR) approach:

- TP (True Positive) is the number of instances correctly classified as the class of interest;
- TN (True Negative) is the number of instances correctly classified as not belonging to the class of interest;
- FP (False Positive) is the number of instances from other classes incorrectly classified as the class of interest;
- FN (False Negative) is the number of instances of the class of interest incorrectly classified as another class.

In addition, Precision was computed to quantify the proportion of correctly predicted instances for each class and is given by:

(2) $$Precision=\frac{TP}{TP+FP}$$

Recall was employed to assess the model’s ability to identify all instances of severity class within the dataset and is formulated as follows:

(3) $$Recall=\frac{TP}{TP+FN}$$

Finally, the F1-score provides a harmonic mean of precision and recall to balance both aspects:

(4) $$F1\text{-}score=2\times \frac{Precision\times Recall}{Precision+Recall}$$

AUC (Area Under the ROC Curve), instead measures the ability of the model to discriminate between classes.

To handle multi-class tasks, the following two different averaging strategies are employed:

- Macro-average: computes the unweighted mean of each metric across all classes, treating all classes equally regardless of their frequency. This highlights performance on minority classes.
- Weighted-average: computes the mean of each metric weighted by the number of samples per class, emphasizing the influence of the majority class.

Additionally, the Quadratic Weighted Kappa (QWK) is used as an ordinal-aware metric to assess agreement between predicted and true labels in tasks with ordered classes. QWK accounts for the degree of disagreement between classes, giving higher penalties for predictions that are farther from the true class. Unlike accuracy, QWK considers not only whether a prediction is correct, but also how large the misclassification is along the ordered scale. This makes it particularly suitable for evaluating classification problems with ordinal labels and imbalanced class distributions.

### 3.3. Grad-CAM Explainability

To enhance the explainability of the developed models, we resort to the Grad-CAM technique. This approach generates activation maps that highlight the regions of the input retinal images most influential in the model decision-making process, allowing the assessment of whether these regions correspond to clinically relevant retinal features associated with DR.

Furthermore, a comparative analysis of the Grad-CAM maps generated by the different architectures was conducted to investigate the effect of architectural design choices, as well as training and fine-tuning strategies, on the models’ ability to focus on informative image regions. This comparison provided additional insights into the consistency and reliability of the learned representations across models.

## 4. Experimental Analysis

In this section, we present and discuss the experimental analysis results, aimed to show the effectiveness of the proposed network, i.e., FGNet, for explainable DR detection.

### 4.1. Dataset

The APTOS 2019 Blindness Detection dataset, freely accessible for research via the Kaggle platform (https://www.kaggle.com/c/aptos2019-blindness-detection accessed on 1 March 2025), was used in the experimental analysis to show the effectiveness of the proposed method. The dataset consists of 3662 images of the fundus with related annotations, provided by the Asia Pacific Tele-Ophthalmology Society (https://asiateleophth.org/ accessed on 1 March 2025).

**Figure 2:**
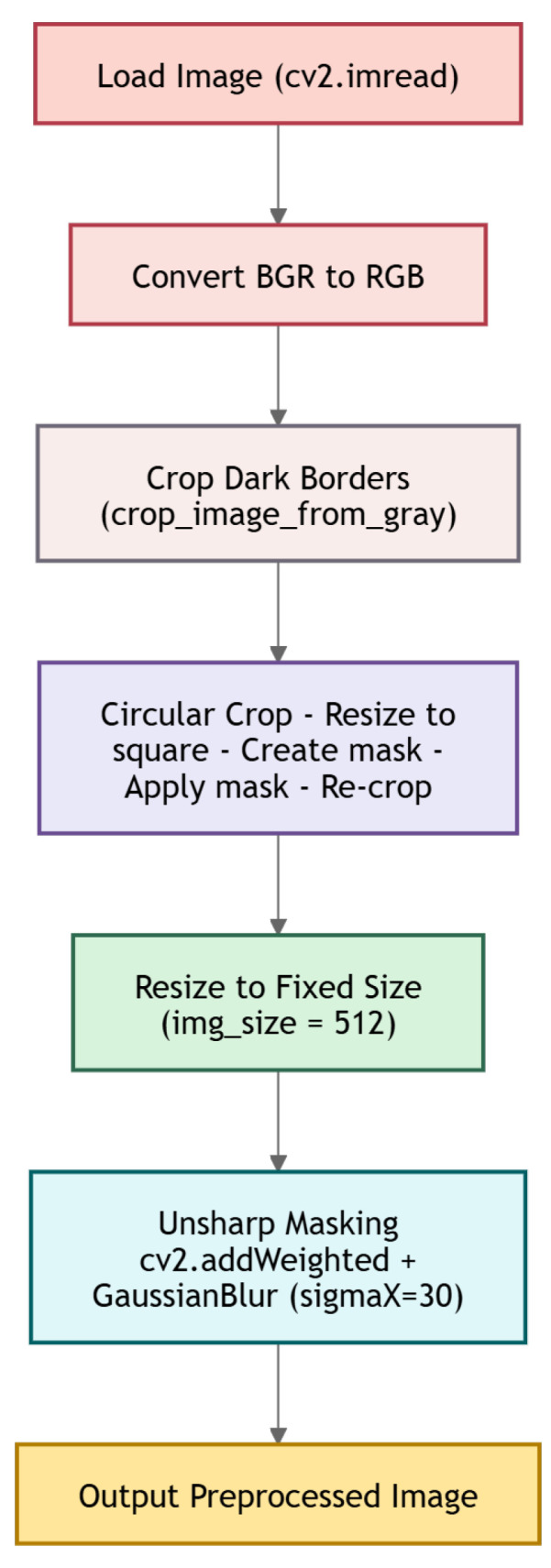
The several steps related to image preprocessing.

Each image was labeled by a clinician from the Asia Pacific Tele-Ophthalmology Society according to the following categories: No DR (healthy retina), Mild DR, Moderate DR, Severe DR, and Proliferative DR. The distribution of images across these categories is reported in Table 2. For dataset splitting, we consider a random seed equal to 42. We also applied a preprocessing, i.e., image cropping and a Gaussian blur filter. In detail, with regard to preprocessing, first, images were loaded in RGB format and dark borders were removed using a grayscale-threshold-based cropping strategy (tolerance=7). A circular crop centered on the fundus was then applied to eliminate peripheral artifacts and background regions. The cropped images were resized to 512×512 pixels. Finally, contrast was enhanced using an unsharp masking technique inspired by Ben Graham, implemented as a weighted combination of the original image and a Gaussian-blurred version (σ=30), in order to emphasize retinal structures while preserving lesion-level details. Figure 2 shows the steps we considered for image preprocessing. All images from the publicly released dataset were included in the training split without any removals or augmentation.

More details about the preprocessing implementation are provided in Listing A1 in Appendix A, showing the Python code developed by authors for image preprocessing.

For neural network training and evaluation, the dataset was partitioned into training, validation, and test subsets following an 80 (training)–10 (validation)–10 (test) split, as shown in Table 3. Data augmentation was applied exclusively to the training set after the split, to help balance the class distribution, increase the number of images, and improve evaluation robustness. In particular, data augmentation included horizontal and vertical flips, as applied in other retinal fundus image studies [20] and were generated using the Keras ImageDataGenerator library. The augmented samples were used only during training, resulting in an effective training set size of 7107 images, while validation (364 images) and test (369 images) sets consisted solely of non-augmented images.

**Figure 3:**
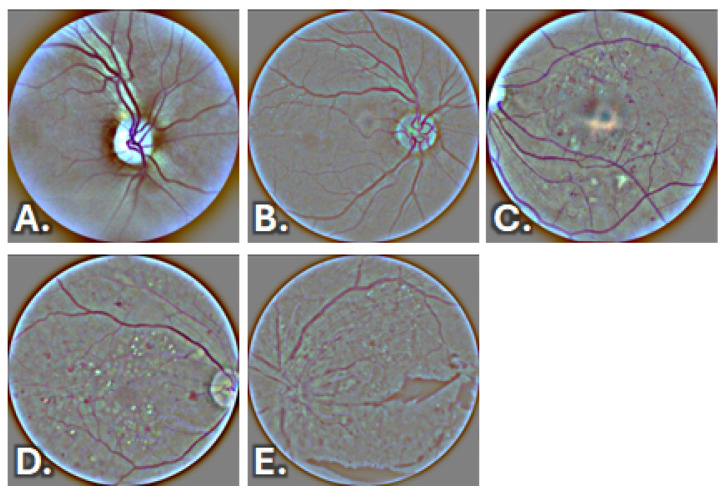
Representative fundus images showing progressive stages of diabetic retinopathy: (**A**) No DR, normal retinal appearance; (**B**) Mild DR, presence of microaneurysms; (**C**) Moderate DR, intraretinal hemorrhages and hard exudates; (**D**) Severe DR, widespread microvascular lesions; (**E**) Proliferative DR, fibrovascular proliferation and neovascular changes.

Figure 3 illustrates representative pre-processed fundus images corresponding to the different stages of DR. All images used in this study are publicly available at the following link: https://drive.google.com/drive/folders/1vnSb6IM7on4GXTA2_vlxrW7_NqJOw8Gt?usp=sharing (accessed on 13 April 2026).

In No DR images, as shown in Figure 3A, no microvascular lesions typical of diabetic retinopathy are visible. Specifically, there is no evidence of microaneurysms, intraretinal hemorrhages, hard or cotton-wool exudates, signs of macular edema, or vascular abnormalities. The optic disc appears normal, with sharp margins and no signs of edema. Retinal vascularization shows a regular caliber and course, and the overall appearance of the retinal fundus is well preserved.

Figure 3B illustrates an example of Mild DR. The fundus image shows a generally well-preserved retina with the presence of a limited number of small punctate hyperreflective lesions scattered across the posterior pole and along the vascular arcades, consistent with microaneurysms and/or minimal intraretinal hemorrhages. The optic disc remains normal, with well-defined margins and no evidence of edema. Retinal vessels maintain an overall normal appearance, without signs of venous beading, intraretinal microvascular abnormalities, or neovascularization. The macular region does not show clear signs of clinically significant edema or extensive hard exudate deposition.

An example of Moderate DR is shown in Figure 3C. The fundus image reveals more widespread microvascular abnormalities involving the posterior pole, characterized by numerous microaneurysms and intraretinal hemorrhages diffusely distributed across the macular and perimacular regions. Multiple hard exudates are visible, often clustered around the macula, indicating increased vascular permeability and lipid deposition. The optic disc remains clearly delineated, with sharp margins and no evidence of disc edema. Retinal vessels exhibit mild abnormalities in caliber and course; however, no venous beading, intraretinal microvascular abnormalities, or neovascularization are observed. The macular region shows signs of involvement that are not suggestive of proliferative disease.

Figure 3D presents an example of Severe DR. The fundus image demonstrates extensive microvascular damage across the posterior pole, with a high density of microaneurysms and intraretinal hemorrhages distributed over multiple retinal quadrants. Numerous hard exudates are present, particularly in the macular and perimacular regions, reflecting marked vascular leakage and advanced retinal involvement. Retinal vessels show abnormal features, including increased tortuosity and irregular caliber, indicative of significant microvascular impairment. The optic disc remains visible with relatively preserved margins, and no definite signs of neovascularization are detected, supporting the classification of severe non-proliferative diabetic retinopathy.

Finally, in Figure 3E, there is an example of Proliferative DR. The fundus image reveals extensive and advanced retinal pathology, characterized by widespread fibrovascular proliferation and disorganization of the retinal architecture. Large areas of fibrovascular tissue are evident, particularly in the posterior pole, consistent with ischemia-driven neovascular processes. Normal retinal layering is markedly disrupted, with tractional changes and retinal distortion caused by fibrovascular membranes. Retinal vessels appear irregular and distorted, with abnormal branching patterns consistent with neovascularization. The macular region is severely involved, and the overall appearance reflects end-stage disease, clearly distinguishing PDR from severe non-proliferative forms.

### 4.2. Experimental Setup

Several CNN architectures were employed as discussed in the previous section, and a novel custom architecture, named FGNet, was specifically designed for DR classification. All experiments were conducted using the TensorFlow v2.18.1 libraries and Python 3.9 [21] as programming language. Data augmentation was selectively applied to the training set, excluding the “No DR” subset, to enhance sample diversity and address class imbalance. Conversely, validation and test sets included only original i.e., non-augmented data.

A systematic hyperparameter tuning process was conducted using a grid search method to determine the most suitable configuration for each CNN model. The optimization focused primarily on learning rate and batch size selection. Learning rates of 0.01, 0.001, and 0.0001 were investigated following a logarithmic scale to ensure a trade-off between training stability and convergence speed. Batch sizes of 16 and 32 were evaluated in order to balance computational efficiency with gradient estimation stability. To ensure a consistent comparison across models, all experiments share identical data splits and augmentation strategies applied across all evaluated architectures.

#### 4.2.1. State-of-the-Art CNNs

To contextualize the performance of the proposed FGNet architecture, a set of CNNs was employed as comparative references. In particular, VGG16, StandardCNN, ResNet, CustomCNN, EfficientNet, MobileNet, and DenseNet were selected and trained on the same dataset to establish performance baselines. These architectures consider different depths, parameter efficiency, and feature extraction strategies.

By training these networks under the same experimental conditions applied to FGNet, it is possible to directly compare learning behavior, convergence stability, and classification performances. The CNN models reported in this section were trained from scratch.

#### 4.2.2. Fine-Tuning of VGG16, DenseNet and ResNet50

Unlike the previous setup, the following models are initialized with pre-trained weights and subsequently fine-tuned on the target dataset. For the fine-tuning experiments, the VGG16 architecture was employed as a feature extractor. Its convolutional backbone was loaded by excluding the final fully connected layers (include_top=False), thereby retaining only the stages responsible for feature extraction. This initialization allows the model to leverage robust low- and mid-level visual representations learned from large-scale data.

During the initial training phase, all layers of the VGG16 backbone were frozen to prevent updates to the pre-trained parameters. This strategy was adopted to preserve generic visual features and to reduce the risk of overfitting, particularly given the limited size of the fundus image dataset. Subsequently, a fine-tuning phase was performed by unfreezing the layers of the last convolutional block, allowing task-specific feature refinement while maintaining training stability for diabetic retinopathy classification.

On top of the convolutional base, a custom classification head was added to adapt the network to the target task. The classifier consisted of four fully connected layers, with the first three containing 512, 256, and 128 neurons, respectively. This progressive reduction in dimensionality was designed to enable hierarchical feature abstraction while controlling model complexity. Batch normalization layers were incorporated within the classification head to mitigate internal covariate shift, leading to improved convergence behavior and enhanced generalization performance.

For the DenseNet-based experiments, the DenseNet architecture pre-trained on ImageNet was adopted as the convolutional backbone. The model was loaded without the final fully connected layers (include_top=False), preserving only the densely connected convolutional blocks responsible for hierarchical feature learning. The use of pre-trained weights allowed the network to leverage rich visual representations learned from large-scale natural image datasets.

Initially, all layers of the DenseNet backbone were frozen to avoid updating pre-trained parameters and to limit overfitting during early training stages. In the subsequent fine-tuning phase, the layers of the fifth dense block were unfrozen, enabling the model to adapt high-level features to the classification of diabetic retinopathy from fundus images while retaining the benefits of transfer learning.

A custom classification head was appended to the frozen backbone, consisting of four fully connected layers with 512, 256, and 128 neurons in the first three layers, respectively. This architecture supports effective feature compression while preserving discriminative capability. Batch normalization was applied within the classification head to improve gradient flow and promote stable optimization during training.

For the ResNet50 experiments, the ResNet50 architecture pre-trained on the ImageNet dataset was employed as the convolutional backbone. The network was loaded by excluding the final fully connected layers (include_top=False), thereby retaining the residual convolutional blocks responsible for deep feature extraction. Pre-trained ImageNet weights were used to capitalize on robust residual feature representations.

During the initial training stage, all layers of the ResNet50 backbone were frozen to preserve the integrity of the learned residual features and to reduce overfitting. Fine-tuning was subsequently carried out by unfreezing the layers of the fifth residual block, allowing the model to specialize higher-level representations for diabetic retinopathy classification from fundus images.

To adapt the backbone to the target task, a custom classification head was added, composed of four fully connected layers with 512, 256, and 128 neurons in the first three layers, respectively. This gradually decreasing structure facilitates feature refinement while maintaining model efficiency. Batch normalization layers were incorporated within the classification head to stabilize training dynamics, improve convergence, and enhance overall optimization reliability.

### 4.3. Quantitative Analysis

#### 4.3.1. Results of FGNet

The performance of the proposed FGNet model was evaluated on the APTOS 2019 dataset using multi-class classification metrics since the task involves five diabetic retinopathy severity levels.

The model was trained using a batch size of 16, a learning rate of 0.01, and an input image size of 110×110×3, with a loss value of 0.8435. To better understand the model behavior across the different severity levels, the confusion matrix and per-class metrics are reported in Table 4 and Table 5. In particular, the per-class performance metrics were computed using the OvR strategy, in which the class of interest is treated as the positive class, and all other classes are considered negative. This approach allows for evaluating each class independently in a multi-class setting.

Furthermore, considering the class imbalance, multiple averaging strategies were used to provide a comprehensive evaluation of the model performance. The weighted accuracy, precision, recall, F1-score, and AUC are 0.8841, 0.7240, 0.7073, 0.7160, and 0.8161, respectively. As for the macro-averages, instead, the accuracy, precision, recall, F1-score, and AUC are 0.8829, 0.5285, 0.5460, 0.5356, and 0.7362, respectively. Note that both macro-averages and weighted-averages for accuracy, precision, recall, F1-score, and AUC are computed from the OvR-based metrics.

The model performs very well on the majority class (No DR) with a precision of 0.9429 and a recall of 0.9116, while performance is lower on minority classes such as Severe DR and Proliferative DR, reflecting the class imbalance of the APTOS 2019 dataset.

To better reflect clinically meaningful agreement between predicted and true DR severity, we also report the QWK of 0.7554, an ordinal-aware metric used in DR grading and crucial for imbalanced datasets [22].

Overall, these results indicate that FGNet provides strong performance for the majority class (No DR) and achieves good overall ordinal agreement as measured by the QWK, while performance on minority classes is lower, reflecting the class imbalance in the dataset.

#### 4.3.2. State-of-the-Art CNN Results

Table 6 presents a detailed comparative analysis of the performance of the CNN architectures evaluated under different training configurations. It is important to note that the goal of this comparison is not to enforce identical hyperparameter configurations across all architectures, but rather to evaluate how different models perform under tuned conditions within the same search space. The idea is to reflect real-world deployment scenarios. This experimental assessment aims to investigate how architectural design choices, training hyperparameters, and input image resolution jointly influence classification performance in the considered retinal disease classification task.

Table 6 is structured into two main sections. The Input Parameters section reports the training settings adopted for each experiment, including the number of epochs, batch size, learning rate, and input image size. In particular, different image resolutions were also tested to evaluate their impact on feature extraction quality and model generalization. This analysis allows assessing whether higher-resolution inputs provide a measurable performance benefit compared to smaller images, while also considering computational efficiency. The Metrics section summarizes the resulting performance in terms of accuracy, loss, precision, and recall, which are standard quantitative indicators for multiclass classification problems.

For each architecture, multiple training configurations are reported to capture the sensitivity of the models to hyperparameter variations. Highlighted rows indicate the best-performing configuration for each network, identified by maximizing accuracy, precision, and recall while minimizing the loss value. This visual emphasis facilitates the identification of optimal settings and supports a direct comparison across different models.

The results show that VGG16 consistently underperforms across all tested configurations, exhibiting low accuracy and null precision and recall, suggesting difficulties in adapting to the dataset under the evaluated training strategies. In contrast, the StandardCNN demonstrates stable and competitive performance, with improvements observed when increasing the number of epochs and adopting moderate batch sizes. These findings indicate that simpler architectures can effectively capture relevant retinal features when appropriately tuned.

ResNet exhibits variable behavior across configurations, with performance strongly influenced by batch size and learning rate. While certain setups yield reasonable accuracy and precision, overall performance remains less consistent compared to lighter architectures. The CustomCNN achieves a balanced performance across metrics, with its best configuration reaching competitive accuracy, precision, and recall values, highlighting the effectiveness of a tailored architecture designed specifically for the target task.

EfficientNet shows limited performance in this experimental setting, with high loss values and low accuracy in most configurations, suggesting sensitivity to the dataset size and training conditions. MobileNet, on the other hand, achieves solid and consistent results, confirming its suitability for efficient image classification tasks where a favorable trade-off between performance and computational cost is required.

Among all evaluated models, DenseNet achieves the highest overall accuracy and recall in its best configuration, indicating a strong capability to extract discriminative features from retinal images. However, this performance is also associated with higher computational demands, emphasizing the trade-off between accuracy and efficiency.

Overall, Table 6 highlights that custom-designed architectures can perform competitively with deeper, more complex CNNs, providing valuable insights for the selection of an appropriate model for DR classification.

#### 4.3.3. Results After Fine-Tuning of VGG16, DenseNet and ResNet50

Table 7 summarizes the classification performance of three fine-tuned convolutional neural network architectures (i.e., VGG16, DenseNet, and ResNet50) evaluated under selected training configurations. For each model, the number of training epochs, batch size, and learning rate are reported together with the corresponding performance metrics, including accuracy, loss, precision, and recall.

All models were trained using the same learning rate and batch size to ensure a fair comparison and to isolate the effect of architectural differences on classification performance. The reported results highlight how different network designs influence the balance between precision and recall when applied to retinal disease classification.

Among the evaluated architectures, DenseNet achieves the highest overall accuracy and the lowest loss, indicating superior generalization capability under the adopted training configuration. Its balanced precision and recall values suggest an effective trade-off between correctly identifying pathological cases and minimizing false positives.

VGG16 demonstrates competitive accuracy and high precision, but a comparatively lower recall, indicating a tendency to correctly classify positive predictions while missing a portion of pathological instances. ResNet50 exhibits the highest precision among the evaluated models; however, this comes at the cost of reduced recall and overall accuracy, suggesting a conservative classification behavior that favors confidence over sensitivity.

Overall, the results reported in Table 7 emphasize the impact of architectural design on classification outcomes, even under identical training settings. The comparative analysis provides insight into the strengths and limitations of each fine-tuned model and supports the selection of DenseNet as the most balanced architecture for the considered retinal disease classification task.

### 4.4. Qualitative Analysis

To qualitatively interpret the models’ predictions, Grad-CAM heatmaps were generated to highlight the regions of the images that contributed most to the predictions, providing a visual assessment of the network’s attention patterns relative to retinal image structures.

The resulting heatmaps were inspected to determine whether the network consistently focused on visually coherent anatomical regions across different classes and correctly classified cases. Although Grad-CAM provides a coarse and qualitative approximation of model attention, the analysis offers insights into the spatial patterns contributing to the network’s predictions.

Images were grouped and displayed according to their corresponding stage of DR classes, enabling direct qualitative comparison of the models’ attention patterns and predictive behavior across different severity levels. The visualizations indicate the regions that most strongly contribute to the classification.

To reduce subjectivity in the interpretation of the heatmaps, examples were selected from the test set to include both correctly classified and misclassified cases across all DR severity levels. The displayed images are representative examples and do not constitute an exhaustive evaluation of all test samples.

Due to the lack of pixel-level lesion annotations, a formal quantitative localization assessment was not feasible. Consequently, the analysis remains qualitative and exploratory, and no lesion-level validation was performed. Moreover, it is important to emphasize that the Grad-CAM visualizations were not reviewed by an independent clinical expert, and no formal ophthalmological validation was conducted to verify whether the highlighted regions correspond to established DR biomarkers (e.g., microaneurysms, hemorrhages, exudates, or neovascularization). Therefore, the observed activation patterns should be interpreted as qualitative indicators of model attention rather than confirmed lesion-level localization. Future work will include expert-based assessment and, where available, lesion-level annotations to enable quantitative clinical validation.

Overall, differences emerge among the evaluated architectures. FGNet demonstrates relatively stable activation patterns across all classes, with attention often focused on anatomically coherent regions such as the optic disc, peripapillary area, and other retinal structures. While some activations overlap with regions exhibiting visible structural alterations, these observations do not constitute formal evidence of lesion-level localization. Importantly, this behavior is achieved without relying on large-scale pretraining or extensive fine-tuning, suggesting that the task-specific architectural design can capture discriminative retinal features while providing qualitative interpretability insights.

In comparison, pretrained architectures without fine-tuning, particularly VGG16, generally exhibit weak, diffuse, or non-informative attention maps, indicating limited adaptability of generic features to the retinal domain. DenseNet and ResNet without fine-tuning show partial localization but tend to rely on broader, less specific contextual cues.

Fine-tuning substantially improves the spatial coherence and stability of the activation maps across pretrained models. After fine-tuning, VGG16, DenseNet, and ResNet progressively shift their attention toward more anatomically coherent retinal regions, with reduced spurious activations and increased consistency across disease classes. Among these, the fine-tuned ResNet model consistently exhibits highly focused, stable, and anatomically coherent activation patterns, suggesting more structured attention behavior during prediction.

**Figure 4:**
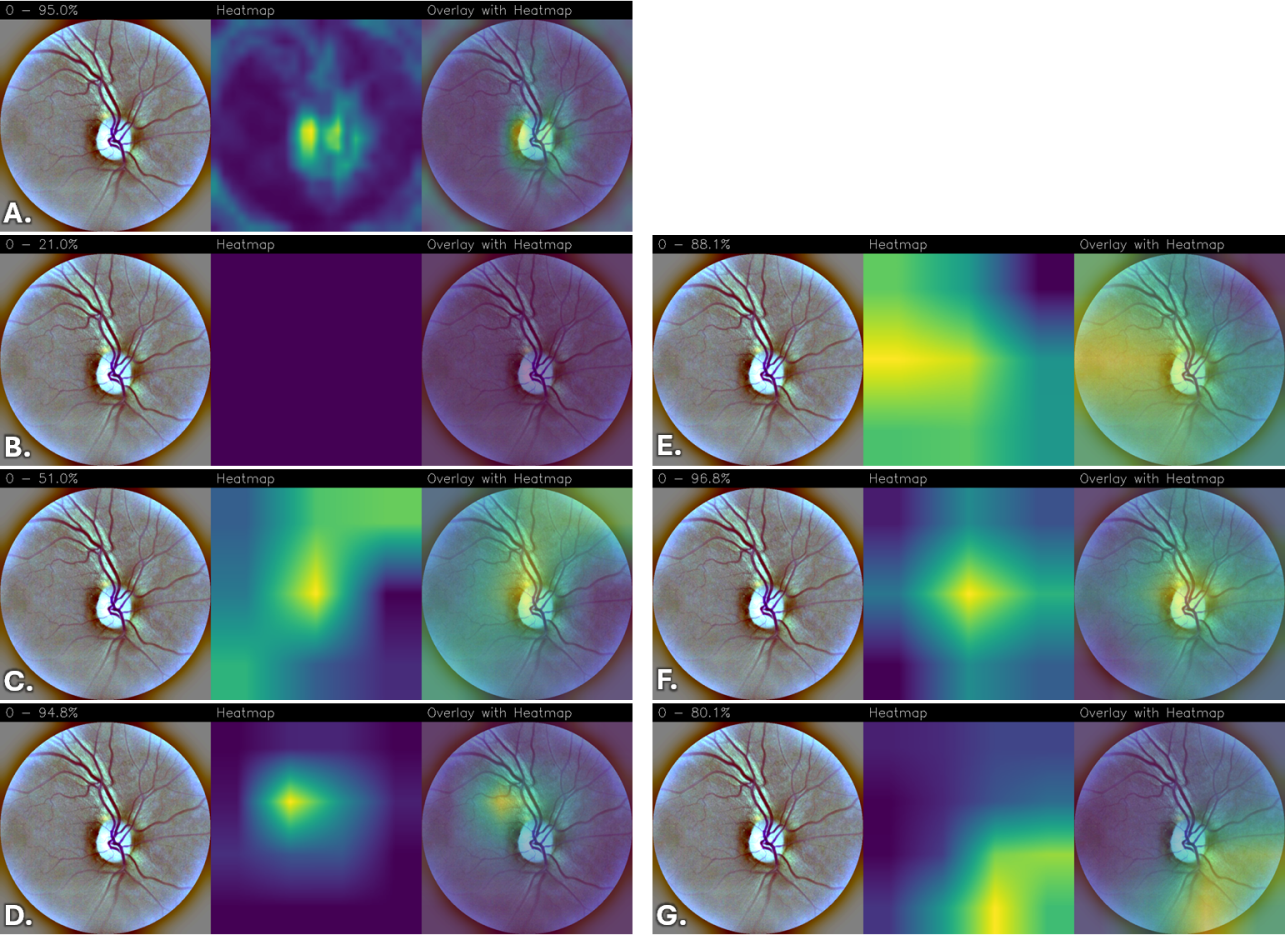
Grad-CAM visualizations for “No DR” across different convolutional neural network architectures. For each model, the original retinal fundus image, the corresponding Grad-CAM heatmap, and the heatmap overlaid on the original image are shown. (**A**) FGNet, (**B**) VGG16, (**C**) DenseNet, (**D**) ResNet, (**E**) VGG16 with fine-tuning, (**F**) DenseNet with fine-tuning, and (**G**) ResNet with fine-tuning.

Figure 4 reports the Grad-CAM visualizations for “No DR” obtained from the different convolutional neural network architectures, highlighting the image regions that contribute most to the final prediction. In Figure 4A, corresponding to the FGNet model, the activation is highly localized and predominantly concentrated on the optic disc region. The heatmap shows a strong and spatially confined response, indicating that the model bases its “No DR” decision on anatomically coherent retinal structures. However, optic-disc–centered activation does not correspond to DR-specific lesions and may reflect reliance on structural reference regions rather than explicit pathological markers.

Conversely, the VGG16 model (Figure 4B) exhibits almost no discriminative activation, as reflected by the nearly uniform and low-intensity heatmap. This behavior suggests that pretrained VGG16 features, when not adapted to the retinal domain, are insufficient to capture class-specific patterns, resulting in weak and poorly localized attention.

The DenseNet model (Figure 4C) displays a broader and more diffuse activation pattern. While partial emphasis on the optic disc is still observable, the attention spreads across a wider retinal region, indicating reliance on global contextual information rather than sharply localized discriminative features. Such diffuse patterns may suggest dependence on global texture or structural cues rather than lesion-specific regions. A similar trend is observed for the non-fine-tuned ResNet model (Figure 4D), which shows a more centralized activation near the optic disc but still with noticeable diffusion toward less relevant regions.

A marked improvement in spatial focus is evident after fine-tuning. The fine-tuned VGG16 model (Figure 4E) demonstrates a clear shift toward more anatomically structured activation patterns, with stronger and more coherent responses. This confirms the importance of fine-tuning in adapting high-level convolutional representations to the specific characteristics of retinal fundus images.

Similarly, the fine-tuned DenseNet architecture (Figure 4F) produces a more structured heatmap, characterized by a strong response at the optic disc and smoother spatial transitions to surrounding regions.

Finally, the fine-tuned ResNet model (Figure 4G) shows one of the most focused and anatomically consistent activation patterns among all evaluated configurations. The attention is tightly concentrated on the optic disc and nearby vascular structures, with minimal peripheral activation, indicating spatially stable attention behavior. While anatomically plausible, this pattern does not constitute formal evidence of lesion-level localization and should be interpreted as qualitative insight into model behavior rather than verified pathological detection.

**Figure 5:**
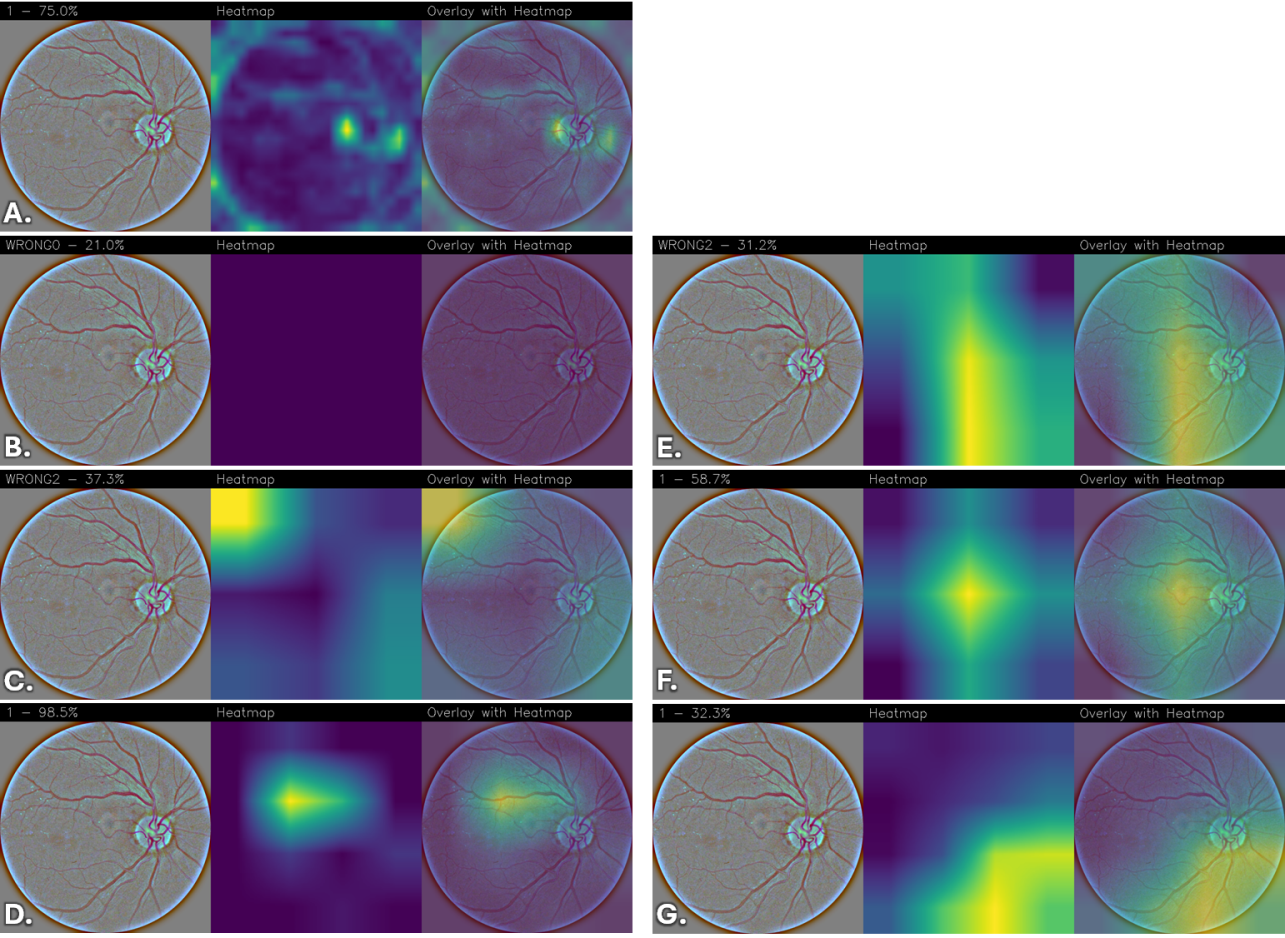
Grad-CAM visualizations for “Mild DR” across different convolutional neural network architectures. For each model, the original retinal fundus image, the corresponding Grad-CAM heatmap, and the heatmap overlaid on the original image are shown. (**A**) FGNet, (**B**) VGG16, (**C**) DenseNet, (**D**) ResNet, (**E**) VGG16 with fine-tuning, (**F**) DenseNet with fine-tuning, and (**G**) ResNet with fine-tuning.

Figure 5 shows the Grad-CAM visualizations corresponding to “Mild DR” predictions for the evaluated convolutional neural network architectures, highlighting the image regions that most strongly influence the classification outcome. In Figure 5A, corresponding to the FGNet model, the activation map reveals a localized but slightly asymmetric focus near the optic disc, with additional moderate responses extending toward the surrounding peripapillary region. This pattern suggests that FGNet identifies “Mild DR”–related features primarily in proximity to the optic disc while also incorporating nearby contextual information.

Also in this case, the VGG16 model without fine-tuning (Figure 5B) exhibits almost no meaningful activation, consistent with the behavior observed across classes.

In Figure 5C, the DenseNet model shows a widely distributed activation pattern, with pronounced responses located away from the optic disc and extending toward peripheral retinal regions. This diffuse attention indicates that the model relies heavily on global image characteristics rather than class-specific anatomical structures, potentially leading to unstable or less reliable predictions.

The ResNet model (Figure 5D), on the other hand, demonstrates a more centralized activation compared to DenseNet, with a dominant focus in the central retinal area. However, the heatmap still displays a noticeable spread toward adjacent regions, suggesting incomplete localization of “Mild DR”–relevant features in the absence of fine-tuning.

As for “No DR”, a substantial refinement in attention patterns is observed after fine-tuning. The fine-tuned VGG16 model (Figure 5E) exhibits a strong and elongated activation spanning a large portion of the retinal field, with increased emphasis along vascular trajectories and peripapillary regions. While the attention is more pronounced than in the non-fine-tuned version, its spatial extent suggests reliance on broader structural cues rather than strictly localized features.

The fine-tuned DenseNet model (Figure 5F) produces a more coherent and symmetric activation pattern, with a concentrated response near the central retina and smoother attenuation toward the periphery. Compared to its non-fine-tuned counterpart, the heatmap is more centered, indicating improved discrimination of “Mild DR”–specific patterns.

Finally, the ResNet model with fine-tuning (Figure 5G) shows the activation primarily distributed across the inferior and temporal retinal regions, with reduced spurious responses elsewhere. The attention pattern suggests that the model captures broader structural or intensity variations associated with this class, while maintaining improved robustness following fine-tuning.

It should be noted that the predictions associated with Figure 5B,C,E have been misclassified, which is reflected in the weak, diffuse, or poorly localized activation patterns observed in the Grad-CAM maps.

**Figure 6:**
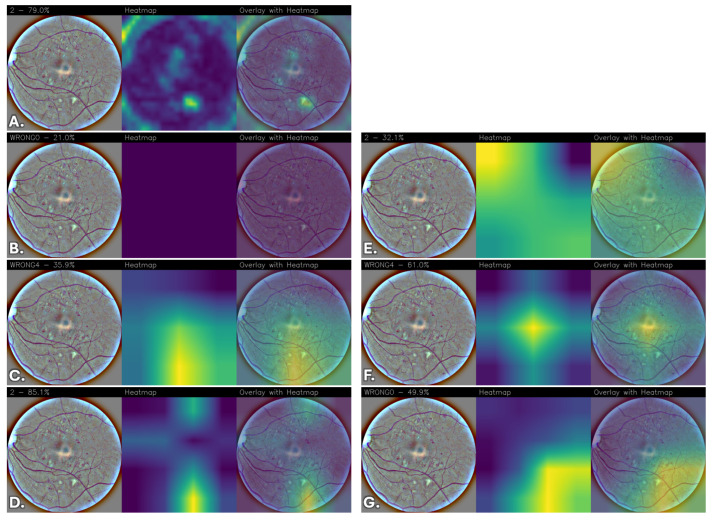
Grad-CAM visualizations for “Moderate DR” across different convolutional neural network architectures. For each model, the original retinal fundus image, the corresponding Grad-CAM heatmap, and the heatmap overlaid on the original image are shown. (**A**) FGNet, (**B**) VGG16, (**C**) DenseNet, (**D**) ResNet, (**E**) VGG16 with fine-tuning, (**F**) DenseNet with fine-tuning, and (**G**) ResNet with fine-tuning.

Figure 6 presents the Grad-CAM visualizations for “Moderate DR” across the different convolutional neural network architectures, highlighting the retinal regions that most influence the classification outcome. In Figure 6A, corresponding to the FGNet model, the activation is moderately localized and primarily concentrated in the central retinal area, with partial emphasis on regions where visible intensity or structural variations are present. The attention appears spatially constrained but less sharply defined than for “No DR”, suggesting that “Moderate DR” is characterized by more heterogeneous visual cues.

The VGG16 model without fine-tuning (Figure 6B) shows an almost complete absence of meaningful activation, as indicated by the uniform and low-intensity heatmap, consistently with the general limitations observed for this architecture without fine-tuning.

Figure 6C shows the heatmap obtained from the DenseNet model and exhibits a broad and diffuse activation pattern extending across a large portion of the retinal field. The attention is not confined to specific anatomical landmarks, indicating reliance on global texture variations rather than localized pathological structures. A similar behavior is observed in the ResNet model (Figure 6D), where activation is spread toward peripheral regions, reflecting incomplete localization of “Moderate DR”–specific features.

The fine-tuned VGG16 model (Figure 6E) shows a stronger activation covering extended retinal regions, particularly around areas exhibiting visible structural or intensity variations. Although the attention remains spatially broad, it is more consistent and structured compared to the non-fine-tuned version.

The DenseNet with fine-tuning model (Figure 6F), instead, produces a more balanced heatmap, characterized by a central concentration of activation with gradual attenuation toward the periphery. This suggests improved discrimination of “Moderate DR”–related features while still leveraging contextual retinal information.

The fine-tuned ResNet model (Figure 6G) demonstrates attention distribution, with activation spanning regions exhibiting diffuse structural variations across the retina. Compared to its non-fine-tuned counterpart, spurious activations are reduced, indicating enhanced robustness and confidence in “Moderate DR” predictions.

With the same image, even in this case, classification associated with Figure 6B,C,F,G have been misclassified.

**Figure 7:**
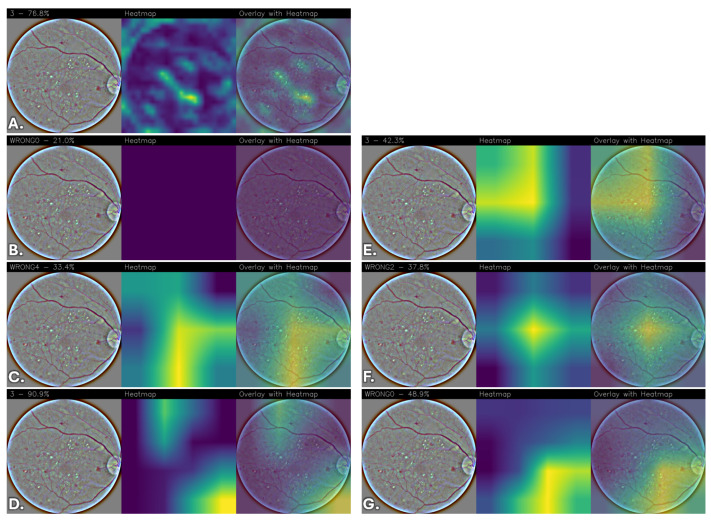
Grad-CAM visualizations for “Severe DR” across different convolutional neural network architectures. For each model, the original retinal fundus image, the corresponding Grad-CAM heatmap, and the heatmap overlaid on the original image are shown. (**A**) FGNet, (**B**) VGG16, (**C**) DenseNet, (**D**) ResNet, (**E**) VGG16 with fine-tuning, (**F**) DenseNet with fine-tuning, and (**G**) ResNet with fine-tuning.

Figure 7 reports the Grad-CAM visualizations for “Severe DR”. In Figure 7A, the FGNet model shows multiple localized activation clusters predominantly distributed across the central and temporal retinal areas. This fragmented attention pattern may reflect the presence of multiple small-scale image variations distributed across the retina, which collectively inform the “Severe DR” prediction.

The VGG16 model without fine-tuning (Figure 7B) again fails to produce meaningful activation, with an almost uniform heatmap, in line with the previously observed behavior.

Figure 7C shows the heatmap obtained from the DenseNet model and exhibits a large, spatially extended activation covering much of the retinal area. While this suggests sensitivity to widespread image variations, the lack of precise localization indicates reliance on global intensity and texture variations rather than discrete pathological markers. A comparable pattern is observed for the ResNet model, Figure 7D, where activation is widespread and partially shifted toward peripheral regions.

The fine-tuned VGG16 model (Figure 7E) shows activation across the retina, aligning with regions exhibiting structural irregularities in the image. Although still spatially extensive, the activation is more consistent and interpretable compared to the non-fine-tuned configuration.

The fine-tuned DenseNet model (Figure 7F), instead, demonstrates a more centralized and symmetric activation pattern, with stronger emphasis on the central retinal region and smoother transitions outward. This reflects improved discrimination of features while maintaining sensitivity to widespread image variations.

Figure 7G represents the ResNet model with fine-tuning and shows an activation predominantly concentrated in regions where structural alterations are visually observable. The reduction in peripheral noise and greater focus on structurally salient retinal regions indicate more stable attention behavior compared to the model without fine-tuning.

Also in this case, as in the previous, in Figure 7B,C,F,G, the predicted labels do not match the ground truth.

**Figure 8:**
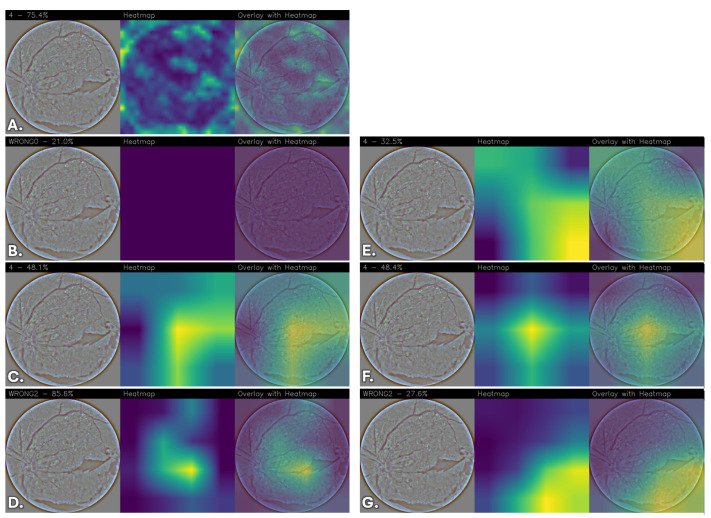
Grad-CAM visualizations for “Proliferative DR” across different convolutional neural network architectures. For each model, the original retinal fundus image, the corresponding Grad-CAM heatmap, and the heatmap overlaid on the original image are shown. (**A**) FGNet, (**B**) VGG16, (**C**) DenseNet, (**D**) ResNet, (**E**) VGG16 with fine-tuning, (**F**) DenseNet with fine-tuning, and (**G**) ResNet with fine-tuning.

Figure 8 illustrates the Grad-CAM visualizations corresponding to “Proliferative DR” predictions. In the FGNet model, shown in Figure 8A, the activation appears moderately localized but fragmented, with salient responses distributed across the central and temporal retinal regions. This dispersed attention pattern suggests that “Proliferative DR” is characterized by complex and heterogeneous visual cues, requiring the model to integrate information from multiple spatially distributed image patterns.

The VGG16 model (Figure 8B) once again shows a lack of meaningful activation, as indicated by the nearly uniform and low-intensity heatmap, consistently with the lack of task-specific adaptation discussed above.

In Figure 8C, the DenseNet model shows a broad and spatially extended activation, with emphasis on a large portion of the retinal field. The attention is not confined to a single anatomical landmark but rather spread across regions exhibiting widespread structural alterations, reflecting the diffuse and spatially extended image patterns observed in advanced disease stages. A similar but slightly more centralized pattern is observed again in the ResNet model (Figure 8D), where activation remains widespread while partially converging toward the central retina.

The fine-tuned VGG16 model (Figure 8E) demonstrates stronger and more coherent activation over extended retinal regions, particularly along areas showing pronounced structural disruption. Although the attention remains spatially broad, it is more consistent and anatomically plausible than in the non-fine-tuned configuration.

On the other hand, the fine-tuned DenseNet model (Figure 8F) produces a more structured and symmetric activation pattern, with a prominent focus near the central retina and smoother attenuation toward peripheral areas. This suggests improved discrimination of “Proliferative DR”–specific features while maintaining sensitivity to the global structural patterns present in the image.

The ResNet model with fine-tuning (Figure 8G) presents a stable and coherent activation predominantly distributed across regions exhibiting widespread structural variations. Compared to its non-fine-tuned counterpart, the heatmap shows reduced noise and improved localization, indicating enhanced robustness and confidence in “Proliferative DR” classification.

In this disease stage, misclassifications occur in the cases depicted in Figure 8B,D,G.

## 5. Conclusions and Future Work

Several convolutional neural network architectures were compared with the aim of identifying the most effective model for DR classification. Specifically, a wide range of CNNs was evaluated, including VGG16, StandardCNN, ResNet, CustomCNN, EfficientNet, MobileNet, DenseNet, and the proposed FGNet architecture. Among these, fine-tuning strategies were applied to the pre-trained DenseNet, ResNet50, and VGG16 models in order to further assess their impact on classification performance.

The obtained results show that DenseNet achieved the highest accuracy, reaching 75%, with well-balanced precision and recall values, making it an effective solution from a purely quantitative perspective. However, FGNet demonstrated competitive numerical performance and exhibited relatively stable and anatomically coherent activation patterns in the Grad-CAM heatmaps. While these visualizations suggest structured spatial attention behavior, they do not constitute formal lesion-level validation. Therefore, explainability findings should be considered qualitative and exploratory rather than conclusive evidence of precise pathological localization.

In contrast, the fine-tuned ResNet50 and VGG16 models, despite achieving high precision values, exhibited very low recall, indicating difficulties in identifying all positive cases. This behavior may limit their practical applicability in screening-oriented settings, where sensitivity is particularly important to reduce the risk of missed pathological cases.

The limitations related to the proposed method concern the qualitative nature of the explainability assessment. The absence of pixel-level lesion annotations prevents objective confirmation of whether activation regions correspond to specific pathological markers. Future work will focus on incorporating datasets with lesion-level annotations and structured clinician-based evaluation protocols to enable quantitative validation of localization plausibility. Additionally, additional explainability techniques such as Grad-CAM++ will be explored with the aim of providing complementary visualization perspectives.

## Figures and Tables

**Table 1:** Comparison of the proposed method with state-of-the-art literature for DR detection and grading. In the case of “N/A” (not applicable), this indicates that, as the paper is a review, a single accuracy value cannot be reported.

Authors	Dataset	Architecture	Method	Accuracy	Explainability
Zhu et al. (2024) [12]	Public DR datasets: EyePACS, APTOS, MESSIDOR-2, IDRiD, DDR, etc.	ResNet, VGGNet, Inception, DenseNet, MobileNet, Transfer Learning, etc.	PRISMA-based survey	N/A	Discussed—future direction
Abushawish et al. (2024) [13]	Public DR datasets: EyePACS, APTOS, MESSIDOR-2, IDRiD, DDR, etc.	26 pre-trained CNN architectures	Survey + transfer learning comparison for DR detection	Detection: 0.78 Grading: 0.75	Grad-CAM
Gulshan et al. (2016) [14]	EyePACS, MESSIDOR-2	Binary classification	Deep CNN	AUC = 0.991 (EyePACS) AUC = 0.990 (MESSIDOR-2)	-
Mercaldo et al. (2023) [10]	EyePACS + APTOS 2019	StandardCNN	Custom CNN	NoDRvsDR: 0.98 NPDRvsPDR: 0.91	Grad-CAM
Correra et al. (2025) [15]	APTOS 2019 Blindness Detection	GLCM features BayesNet, IBk, KStar, LWL	Radiomics ML classifier	0.85	-
Mohanty et al. (2023) [16]	APTOS 2019 Blindness Detection	VGG16 + XGBoost DenseNet121	CNN	1st model: 0.80 2nd model: 0.97	-
This work	APTOS 2019 Blindness Detection	FGNet	Custom CNN	0.75	Grad-CAM

**Table 2:** Distribution of retinal images in the APTOS 2019 Blindness Detection dataset according to their corresponding label.

Class	Number of Images
No DR	1805
Mild	370
Moderate	999
Severe	193
Proliferative	295
**Total**	**3662**

**Table 3:** Distribution of retinal images across training, validation, and testing subsets. The last column reports the effective size of the training set after augmentation. Validation and testing sets contain only original images.

Class	Training	Validation	Testing	Augmented Training
No DR	1444	180	181	Not Augmented
Mild DR	296	37	37	1396
Moderate DR	799	99	101	1448
Severe DR	154	19	20	1420
Proliferative DR	236	29	30	1399
**Total**	2930	364	369	7107

**Table 4:** Confusion matrix for FGNet predictions. Shaded cells indicate correct predictions for each class.

Actual Classes	Predicted Classes				
	No DR	Mild DR	Moderate DR	Severe DR	Proliferative DR
No DR	165	9	7	0	0
Mild DR	4	21	6	2	4
Moderate DR	6	8	59	9	19
Severe DR	0	2	5	8	5
Proliferative DR	0	3	15	4	8

**Table 5:** Performance metrics for FGNet.

Class	Accuracy	Precision	Recall	F1-Score	AUC
No DR	0.9295	0.9429	0.9116	0.9270	0.9292
Mild DR	0.8970	0.4884	0.5676	0.5250	0.7507
Moderate DR	0.7967	0.6413	0.5842	0.6114	0.7305
Severe DR	0.9268	0.3478	0.4000	0.3721	0.6785
Proliferative DR	0.8645	0.2222	0.2667	0.2424	0.5920
Macro-average	0.8829	0.5285	0.5460	0.5356	0.7362
Weighted-average	0.8841	0.7239	0.7103	0.7142	0.8161

**Table 6:** Performance comparison of different CNN architectures under various training configurations. Best results are highlighted.

Architecture	Input Parameters				Metrics			
	Epochs	Batch Size	Learning Rate	Image Size	Accuracy	Loss	Precision	Recall
VGG16	10	16	0.01	224 × 3	0.49051	1.58849	0	0
	20	32	0.0001	224 × 3	0.27371	1.60076	0	0
	25	32	0.0001	224 × 3	0.27371	1.59849	0	0
	20	16	0.001	224 × 3	0.27371	1.59797	0	0
	5	16	0.001	110 × 3	0.27371	1.61027	0	0
StandardCNN	20	16	0.001	110 × 3	0.71816	1.36318	0.72727	0.69377
	20	16	0.001	224 × 3	0.71274	1.24530	0.73487	0.69106
	30	16	0.001	110 × 3	0.72358	1.45234	0.73938	0.77317
	30	32	0.001	110 × 3	0.72867	1.52624	0.73650	0.70461
	20	32	0.001	110 × 3	0.69919	1.25243	0.72093	0.67209
ResNet	5	16	0.001	110 × 3	0.62331	1.02109	0.75833	0.49322
	5	32	0.001	224 × 3	0.41192	1.39986	0.88028	0.33875
	5	32	0.0001	110 × 3	0.44173	2.22867	0.44058	0.41192
	10	16	0.001	110 × 3	0.59349	2.16406	0.60906	0.58266
	10	32	0.001	110 × 3	0.70461	1.15239	0.72174	0.67480
CustomCNN	10	16	0.001	110 × 3	0.68294	1.06777	0.72321	0.65854
	10	32	0.001	110 × 3	0.67209	1.10165	0.73602	0.64228
	10	16	0.001	224 × 3	0.71816	1.05019	0.73199	0.68835
	20	16	0.001	110 × 3	0.70190	1.38602	0.72414	0.68293
	20	32	0.001	110 × 3	0.72899	1.23466	0.75284	0.71816
EfficientNet	10	16	0.001	110 × 3	0.47696	2.80548	0.47826	0.47696
	10	32	0.001	110 × 3	0.40921	3.26149	0.44781	0.36043
	5	16	0.001	224 × 3	0.10027	3.90357	0.10027	0.10027
	5	16	0.001	110 × 3	0.28726	1.51797	0.28655	0.26558
	10	16	0.0001	110 × 3	0.44444	1.63100	0.42738	0.33875
MobileNet	5	16	0.001	110 × 3	0.53930	1.57885	0.55425	0.51219
	10	16	0.001	110 × 3	0.54471	1.87781	0.56250	0.53658
	10	32	0.001	110 × 3	0.70732	1.23371	0.72159	0.68835
	20	16	0.001	110 × 3	0.68564	1.83091	0.70112	0.68022
	10	16	0.001	110 × 3	0.71003	1.46738	0.72067	0.69919
DenseNet	5	16	0.001	110 × 3	0.60163	2.88875	0.73234	0.53387
	5	32	0.001	110 × 3	0.30894	2.57413	0.40161	0.27100
	10	16	0.001	110 × 3	0.47967	5.25609	0.50893	0.46341
	10	16	0.001	110 × 3	0.75068	1.07036	0.75562	0.72899
	10	32	0.001	110 × 3	0.61789	0.94453	0.86441	0.41463

**Table 7:** Performance comparison of fine-tuned pre-trained CNN architectures.

Model	Epochs	Batch Size	Learning Rate	Accuracy	Loss	Precision	Recall
VGG16_ft	5	16	0.001	0.5962	0.9458	0.8944	0.3902
DenseNet_ft	10	16	0.001	0.6477	0.8414	0.7665	0.5339
ResNet50_ft	10	16	0.001	0.5203	1.1679	0.9090	0.3252

## Data Availability

The original dataset used in this study is publicly available at https://www.kaggle.com/c/aptos2019-blindness-detection (accessed on 1 March 2025). The preprocessed dataset generated and used during the current study is publicly available at https://drive.google.com/drive/folders/1vnSb6IM7on4GXTA2_vlxrW7_NqJOw8Gt?usp=sharing (accessed on 13 April 2026).

## Appendix A

**Listing A1.** The Python code snippet for image preprocessing.

## References

[1] Tan T.E., Wong T.Y. (2023). Diabetic retinopathy: Looking forward to 2030. Front. Endocrinol..

[2] Yang Z., Tan T.E., Shao Y., Wong T.Y., Li X. (2022). Classification of diabetic retinopathy: Past, present and future. Front. Endocrinol..

[3] Simó R., Hernández C. (2022). New insights into treating early and advanced stage diabetic retinopathy. Int. J. Mol. Sci..

[4] Lim G., Bellemo V., Xie Y., Lee X.Q., Yip M.Y., Ting D.S. (2020). Different fundus imaging modalities and technical factors in AI screening for diabetic retinopathy: A review. Eye Vis..

[5] Shi C., Lee J., Wang G., Dou X., Yuan F., Zee B. (2022). Assessment of image quality on color fundus retinal images using the automatic retinal image analysis. Sci. Rep..

[6] Celik C., Yücedağ İ (2026). The role of deep learning-based algorithms in retinal image processing: A review. Multimed. Tools Appl..

[7] Li Y., Xia X., Paulus Y.M. (2018). Advances in retinal optical imaging. Photonics.

[8] Mahalakshmi V., Balachandra A., Kanisha B., Chidambaram K. A Deep Learning Approach for Detecting Diabetic Retinopathy Using Fundus Images. Proceedings of the International Conference on Internet of Things.

[9] Jain R., Magia D., Joy J.E. Diabetic Retinopathy Detection Using Real-World Datasets of Fundus Images. Proceedings of the International Conference on Data Management, Analytics & Innovation.

[10] Mercaldo F., Di Giammarco M., Apicella A., Di Iadarola G., Cesarelli M., Martinelli F., Santone A. (2023). Diabetic retinopathy detection and diagnosis by means of robust and explainable convolutional neural networks. Neural Comput. Appl..

[11] Montaser E., Shah V.N. (2024). Prediction of incident diabetic retinopathy in adults with type 1 diabetes using machine learning approach: An exploratory study. J. Diabetes Sci. Technol..

[12] Zhu S., Xiong C., Zhong Q., Yao Y. (2024). Diabetic retinopathy classification with deep learning via fundus images: A short survey. IEEE Access.

[13] Abushawish I.Y., Modak S., Abdel-Raheem E., Mahmoud S.A., Hussain A.J. (2024). Deep learning in automatic diabetic retinopathy detection and grading systems: A comprehensive survey and comparison of methods. IEEE Access.

[14] Gulshan V., Peng L., Coram M., Stumpe M.C., Wu D., Narayanaswamy A., Venugopalan S., Widner K., Madams T., Cuadros J. (2016). Development and validation of a deep learning algorithm for detection of diabetic retinopathy in retinal fundus photographs. JAMA.

[15] Correra S., Sorgente V., Cesarelli M., Gargiulo P., Santone A., Mercaldo F. A Method for Diabetic Retinopathy Detection Through Radiomics and Machine Learning. Proceedings of the 2025 IEEE International Conference on Metrology for eXtended Reality, Artificial Intelligence and Neural Engineering (MetroXRAINE).

[16] Mohanty C., Mahapatra S., Acharya B., Kokkoras F., Gerogiannis V.C., Karamitsos I., Kanavos A. (2023). Using deep learning architectures for detection and classification of diabetic retinopathy. Sensors.

[17] Abràmoff M.D., Folk J.C., Han D.P., Walker J.D., Williams D.F., Russell S.R., Massin P., Cochener B., Gain P., Tang L. (2013). Automated analysis of retinal images for detection of referable diabetic retinopathy. JAMA Ophthalmol..

[18] De Fauw J., Ledsam J.R., Romera-Paredes B., Nikolov S., Tomasev N., Blackwell S., Askham H., Glorot X., O’Donoghue B., Visentin D. (2018). Clinically applicable deep learning for diagnosis and referral in retinal disease. Nat. Med..

[19] Di Giammarco M., Vitulli C., Cirnelli S., Masone B., Santone A., Cesarelli M., Martinelli F., Mercaldo F. (2025). Explainable Deep Learning for Breast Cancer Classification and Localization. ACM Trans. Comput. Healthc..

[20] Mishra A., Pandey M., Singh L. (2025). SwinEff-DR: Hybrid Swin Transformer & Efficient Net Architecture for Multi-Scale Diabetic Retinopathy Detection. Informatica.

[21] Abadi M., Agarwal A., Barham P., Brevdo E., Chen Z., Citro C., Corrado G.S., Davis A., Dean J., Devin M. (2015). TensorFlow: Large-Scale Machine Learning on Heterogeneous Systems.

[22] Chilukoti S.V., Shan L., Tida V.S., Maida A.S., Hei X. (2024). A reliable diabetic retinopathy grading via transfer learning and ensemble learning with quadratic weighted kappa metric. BMC Med. Inform. Decis. Mak..

